# Effect of Cladribine on Neuronal Apoptosis: New Insight of In Vitro Study in Multiple Sclerosis Therapy

**DOI:** 10.3390/brainsci10080548

**Published:** 2020-08-13

**Authors:** Maddalena Ruggieri, Concetta Domenica Gargano, Anna Ferretta, Alessia Manni, Antonio Capacchione, Antonio Frigeri, Pietro Iaffaldano, Maria Trojano, Damiano Paolicelli

**Affiliations:** 1 Department of Basic Medical Science, Neuroscience and Sense Organs, School of Medicine, University of Bari Aldo Moro, 70124 Bari, Italy; maddalena.ruggieri@uniba.it (M.R.); garganoimma@gmail.com (C.D.G.); anna.ferretta@uniba.it (A.F.); ali.manni@libero.it (A.M.); antonio.frigeri@uniba.it (A.F.); pietro.iaffaldano@uniba.it (P.I.); maria.trojano@uniba.it (M.T.); 2 Medical Affairs Department, Merck Serono S.p.A., 00176 Rome, Italy; antonio.capacchione@merckgroup.com

**Keywords:** multiple sclerosis, cladribine, apoptosis, neurons

## Abstract

Background: Cladribine (2-CdA) can cross the blood–brain barrier, resulting in inhibition of DNA synthesis and repair and disruption of cellular proliferation in actively dividing lymphocytes. No data on effect on neurons are available. Aim: To study “in vitro” 2-CdA apoptotic effects on neurons in healthy donor and multiple sclerosis patient lymphocytes. Methods: Neuroblastoma cells were co-cultured with lymphocytes, with and without 2-CdA. Results: Apoptosis increased in lymphocytes with 2-CdA; increase was also observed when lymphocytes were cultured with neuronal cells. However, neurons were not affected by 2-CdA for apoptosis. Conclusions: 2-CdA causes peripheral and central lymphocyte death preserving neurons, with a reasonable impact on inflammation and neuroprotection.

## 1. Introduction

Multiple sclerosis (MS) is a chronic neurodegenerative inflammatory disease of the central nervous system (CNS), and it is a cause of non-traumatic disability in young adults [1]. MS involves a complex chain of events with a contribution from several cell types with different immune functions. Cells of the adaptive immune system, autoreactive T and B lymphocytes, are key players in the pathophysiologic processes in the active phase of the disease, above all in the recruitment of cells of the innate immune system. T cells, in particular, play a key role in the pathogenesis of MS. Both CD4+ and CD8+ T cells are present in MS lesions, with CD4+ T cells tending to predominate in acute lesions and CD8+ T cells being more prevalent in chronic lesions [2,3]. Events at the immunologic synapse are central to the activation of autoreactive CD4+ T cells in MS [4,5]. Much of the current understanding of the potential role of CD4+ T cells in MS comes from the animal models simulating features of MS. Experimental autoimmune encephalomyelitis (EAE) is an inflammatory CNS demyelinating disease induced in animals via immunization with myelin proteins or peptides [6], reproducing the clinical and immunological aspect of MS. On the other hand, the decisive role of B cells in MS is endorsed by their contribution to the destruction of myelin by the production of myelin-specific antibodies, and in addition, they can serve as antigen-presenting cells and therefore could play a role in the reactivation of autoreactive T cells in the CNS [7]. B cells are professional antigen-presenting cells (APC): They recognize even low concentrations of antigens specifically and constitutively expressing major histocompatibility complex (MHC) class II and co-stimulatory molecules. This enables B cells to prime T cells and in turn induces their differentiation into memory cells and antibody-producing plasma cells [7,8]. Studies conducted on EAE animal models further confirmed the active involvement of B cells in the pathogenesis of MS, potentially by activating CNS- infiltrating T cells that in turn drive inflammation in the brain and spinal cord [9], thus demonstrating that selective ablation of MHC class II on B cells renders mice resistant to disease induction, showing their substantial contribution as APC to this model.

Specific pairs of chemokine receptors and ligands are known to play pathogenic roles in MS. CX3CL1/CX3CR1; CXCL13/CXCR5; CXCL9/CXCR3; CCL5/CCR1, CCR3, CCR5; CCL2/CCR2; and CCR3 are expressed by astrocytes, macrophages, and microglia within acute and chronic lesions in MS [10]. They can form concentration gradients that attract and activate leukocytes, promoting extravasation, facilitating the trafficking of autoreactive T and B cells across the disrupted blood–brain barrier (BBB) to lesion sites within the CNS where they are reactivated and contribute to demyelination and axonal damage. Leukocyte extravasation through the BBB involves a lot of adhesion molecules, such as vascular cell adhesion molecules (VCAM), intercellular adhesion molecules (ICAM), α4-integrin (VLA-4), and leukocyte functional antigen (LFA-1). In MS, T cells that recognize the specific auto-antigen in the CNS are then reactivated and expanded, clonally amplifying the immune response. These produced cytokines induce the expression of endothelial adhesion molecules and inflammatory mediators, allowing structural changes of the BBB with consequent secondary influx of immune cells, reinforcing the inflammatory response [11]. Understanding the multifaceted pathogenesis of MS provides the basis for potential new immunotherapeutic strategies, using drugs able to cross the BBB [12,13].

Cladribine (2-chloro-2’-deoxyadenosine, 2-CdA), a synthetic purine nucleotide, has shown clinical efficacy in phase III clinical trials in relapsing–remitting MS [14,15]. It is used as therapy against hairy cell leukemia, chronic lymphocytic leukemia, and non-Hodgkin lymphomas [16]. The possible neuroprotective effects of 2-CdA in the CNS have been investigated through the intracerebroventricular (Icv) administration in EAE animal models, highlighting that the clinical defects of EAE mice can be significantly attenuated by 2-CdA, and suggesting that it could afford neuroprotective effects in patients with MS [17]. In the pathogenesis of MS, early lesions of BBB promote T-cell migration to the CNS [18]. Activated T cells penetrate the CNS and contribute to the creation of demyelination foci. 2-CdA is known for its apoptotic effects on peripheral blood and CNS mononuclear cells, especially lymphocytes [9], which physiologically play a protective role in the CNS. T cells are the major cellular component of the cerebrospinal fluid (CSF), and only one percent of the T cells in the periphery has been estimated to be found in the CSF in physiological conditions [19]. Low-level T cell migration in the brain occurs in healthy individuals [20] as the CNS is an immune privileged site where cell trafficking is controlled by the BBB and blood–CSF barrier. CD4+ T cell trafficking is more frequent than CD8+ T cells, leading to a higher CD4/CD8 T cell ratio in CSF compared to blood [19,21]. In the brain, 2-CdA can play different roles; in particular, its ability to pass through the BBB has been well documented [22,23]. Nevertheless, until now, there has been a paucity of data regarding its effects on neurons. The 2-CdA was designed by adding one chlorine atom to deoxyadenosine, making it largely resistant to degradation by ADA. ADA is an intracellular enzyme that degrades deoxyadenosine, which is a purine nucleoside that is required for DNA synthesis, but if it accumulates to high intracellular levels, it inhibits DNA synthesis and leads to lymphocyte depletion by apoptosis.

High intracellular cladribine levels—due to resistance to degradation by ADA—allow cladribine to reduce B and T lymphocyte counts [24]. It has been tested for safety and efficacy and been used either alone or in combination with other cytotoxic agents, for the treatment of several lymphocytic leukemias [25,26,27,28,29]. The 2-CdA acts as a prodrug, and its activity is dependent on the intracellular accumulation of its active triphosphate. The accumulation of active triphosphate deoxynucleotides (CdATPs) interferes with the DNA repair of single-stranded breaks, eventually resulting in cell death. In dividing cells, CdATP can also be incorporated into the DNA, impairing transcription. The 2-CdA causes apoptosis through the caspase system where the cytochrome C and apoptotic protease-activating factor activate caspase-3 and damage DNA: This mechanism interferes with the synthesis and repair of DNA in both resting and dividing lymphocytes [30]. It is believed that 2-CdA induces caspase-dependent apoptosis via mitochondrial and death receptor pathways [25,31]. Data from literature indicate that 2-CdA is a drug widely active on CNS, and for this reason, it was analyzed on different cell types. Kopadze and colleagues demonstrated that 2-CdA inhibits the migration of inflammatory cells into and within the CNS using peripheral blood mononuclear cells isolated from patients and healthy donors [32]. They observed that the inhibitory effect of cladribine was most prominent in CD4+ and CD8+ lymphocytes, but a significant reduction in their migration across the fibronectin layer was also seen for monocytes. This exhibits an effect of 2-CdA on the migratory capacity of mononuclear cells across the BBB into and within the CNS that is considered a key event in the immunopathogenesis of MS. Moreover, it was recently published that 2-CdA is able to modify functional properties of activated microglia. Jorgensen and colleagues demonstrated that in vitro stimulation of these cells with 2-CdA did not affect cell viability, and additionally, 2-CdA potentiated the gene expression of both anti- and pro-inflammatory molecules, such as the anti-inflammatory TNF-R2 [32,33].

To date, there has been a lack of information about 2-CdA effects on neuronal cells, so our aim was to investigate in vitro the possible role of this drug on apoptotic mechanisms and related molecules.

## 2. Materials and Methods

### 2.1. Study Design and Patients

Twenty age- and sex-matched healthy donors (HDs; mean age ± standard deviation [SD], 43.6 ± 20.1 years; 10 female) and 20 MS patients without therapy (mean age ± SD, 45.5 ± 21.4 years; 11 female) were enrolled in this study. Patients enrolled in the study were all treatment-naïve patients affected by relapsing–remitting multiple sclerosis (RR-MS). The drug was prescribed according to the indication granted by the European Medicines Agency (EMA) in the European public assessment report (EPAR) of 2017 (EMA/405631/2017) [34]. Blood sampling was carried out at the time of diagnosis and then regularly as per clinical practice. All patients provided written informed consent before enrollment. The study was conducted in accordance with the International Conference on Harmonization Guidelines for Good Clinical Practice and the Declaration of Helsinki (World Medical Association Declaration of Helsinki (2017) Ethical principles for medical research involving human subjects. Ethics Committee number 5275; prot No 0054862; 11/7/2017; Policlinico, Bari, Italy). The study was performed on behalf of the MS-RUN project (Merck Serono, Geneva, Switzerland).

### 2.2. Peripheral Blood Mononuclear Cell Purification

Lymphocytes of HDs and MS patients (without therapy) were obtained from human peripheral blood mononuclear cells (PBMCs). Human PBMCs were isolated from K3-EDTA-collected venous blood samples by the density gradient centrifugation method using Lymphosep, Lymphocyte Separation Media (Biowest, San Francisco, CA, USA) [20]. PBMCs were cultured in Roswell Park Memorial Institute-1640 (RPMI-1640, Lonza, Basel, Switzerland) medium supplemented with 10% fetal bovine serum (FBS), 100 IU/mL penicillin, and 100 µg/mL streptomycin (Gibco by Thermo Fisher Scientific, MA, USA). Cells were plated (0.5 × 10^6^ cells/well) in 24-well plates at 37 °C in a humid atmosphere containing 5% CO_2_; after 24 h, lymphocytes (non-adherent cells) were collected and seeded on a sterile 24-well plate. Through this method, a pure lymphocyte preparation was obtained, confirmed by May–Grunwald staining.

### 2.3. SH-SY5Y Cell Culture

The SH-SY5Y cells, a human neuroblastoma cell line, were maintained as monolayer cultures in Dulbecco’s modified Eagle’s medium (Gibco by Thermo Fisher Scientific, MA, USA), supplemented with 10% FBS, 100 IU/mL penicillin, and 100 µg/mL streptomycin (Gibco by Thermo Fisher Scientific, MA, USA) at 37 °C in a humid atmosphere containing 5% CO_2_. Cells were plated at a density of 4 × 10^4^/cm^2^ and then used to obtain co-cultures with lymphocytes.

### 2.4. Cladribine Treatment

SH-SY5Y cells were seeded at a density of 4 × 10^4^ cells/cm2 on 24-well multiplates, and then lymphocytes from HDs and from MS (without therapy) patients were added to neuronal cells (1:10 ratio). Lymphocytes and SH-SY5Y co-cultures were exposed to 20 nM 2-CdA for 24 h, to test neuron response to therapy and to autoreactive cells. A total of 20 nM dimethyl sulfoxide (DMSO) was used as control for resuspending-drug medium and 2 mM dithiothreitol (DTT) as the positive control for dead cells.

### 2.5. Western Blot Analysis

Lymphocytes and SH-SY5Y cells were washed with phosphate-buffered saline (PBS) and lysed on ice, with a buffer pH 7.5 (150 mM NaCl, 15 mM MgCl, 1 mM ethylene glycol tetraacetic acid (EGTA), 50 mM HEPES, 10% glycerol, 1% Triton) containing a cocktail of protease inhibitors (Roche, Switzerland) to obtain total proteins. The lysis was performed on ice for 1 h, and then the samples were centrifuged at 22,000× *g* for 45 min. Supernatant protein content was measured with a bicinchoninic acid Protein Assay Kit (Thermo Fisher Scientific, MA, USA). Of total protein samples, 30 µg was separated by 4–15% Tris-Glycine-SDS-PAGE and transferred to nitrocellulose membranes of Trans-Blot^®^ Turbo™ Transfer Pack (#1704158, Bio-Rad, San Francisco, CA, USA). Blots were blocked for 2 h in blocking buffer (PBS buffer, pH 7.4 with 0.2% Tween 20 and 5% nonfat milk) and incubated with primary antibody mouse monoclonal anti-DIABLO (1:1000, SMAC 17 1–87, Thermo Fisher Scientific, MA, USA) and mouse monoclonal anti-Bcl-2 (C-2) (1:200, sc-7382, Santa Cruz Biotechnology, Dallas, TX, USA), overnight at 4 °C. The membranes were washed with TBS/Tween 20 and incubated with peroxidase-conjugated goat anti-mouse secondary antibodies IgG (sc-2005) dilution 1:5000 (Santa Cruz Biotechnology, TX, USA) for 1 h at room temperature. Reactive proteins were revealed with an enhanced chemiluminescent detection system (Bio-Rad, Santa Rosa, CA, USA) and visualized on a Chemidoc imaging system (Bio-Rad, CA, USA). The band intensity was quantitatively determined using Image Lab Software 6.1 (Bio-Rad, CA, USA), and protein level intensity was normalized to Glyceraldehyde 3-phosphate dehydrogenase (GAPDH) expression. Proteins were detected by chemiluminescent detection system (Bio-Rad, CA, USA) and visualized on a Chemidoc imaging system (Bio-Rad, CA, USA). The band intensity was quantitatively determined by densitometric analysis using Image Lab Software 5.2.1 (Bio-Rad Laboratories, CA, USA).), and protein level intensity was normalized to GAPDH expression.

### 2.6. Flow Cytometry on SH-SY5Y

SH-SY5Y cells were exposed to 20 nM 2-CdA for 4, 24, 48, and 72 h. Not treated (controls) and 20 nM DMSO-treated cells were used as negative controls and 2 mM DTT as a positive control of cell death. The percentage of apoptotic events was evaluated by flow cytometry using PE-Annexin V/Dead Cell Apoptosis Kit (Invitrogen by Thermo Fisher Scientific), which identifies apoptotic cells of phenotypically defined subsets by the expression of phosphatidylserine (PS) on the outer side of the plasma membrane. This assay offers the possibility of detecting early phases of apoptosis before the loss of cell membrane integrity through annexin V. Conversely, the SYTOX Green dye is impermeant to live cells and apoptotic cells but stains dead cells with intense green fluorescence by binding cellular nucleic acids. Cells were washed and centrifuged at 425× *g* for 5 min and resuspended in the binding buffer provided by the assay kit at the concentration of ~1 × 10^6^ cells/mL. Cells were incubated with R-PE annexin V and SYTOX Green stain working solution for 15 min at 37 °C in an atmosphere of 5% CO_2_ and then analyzed by flow cytometry. The population was separated into three groups: live cells with only a low level of green and orange fluorescence, apoptotic cells with a high level of orange fluorescence and little green fluorescence, and late apoptotic cells with a high level of green and orange fluorescence. Cells were acquired on CytoFLEX (Beckman Coulter, Brea, CA, USA), and analyzed using CytExpert 2.0 software (Beckman Coulter, CA, USA).

### 2.7. Statistical Analysis

Flow cytometric analysis data are expressed as mean ± SD, and densitometric analysis of protein is expressed as mean ± standard error of the mean of the number of experiments (*n*) indicated in the figure legend. In all the assays, “*n*” refers to the number of independent experiments performed on different cell preparations. Statistically significant differences were computed using Student’s *t*-test analysis or one-way analysis of variance (ANOVA), followed by the Newman–Keuls test.

This sample size of 20 age- and sex-matched healthy donors and 20 MS patients off therapy was considered sufficient, and results were considered as statistically significant at *p* < 0.05. All statistical analysis was performed with GraphPad Prism version 5.00 (GraphPad, San Diego, CA, USA).

## 3. Results

### 3.1. Cladribine Effects on Lymphocyte Cell Cultures and on Lymphocyte and SH-SY5Y Co-Cultures

Proteins from HD and MS single-cultured lymphocytes and lymphocytes co-cultured with SH-SY5Y neuronal cells were analyzed for Smac/DIABLO and Bcl-2 expression after incubation with 2-CdA.

**Figure 1:**
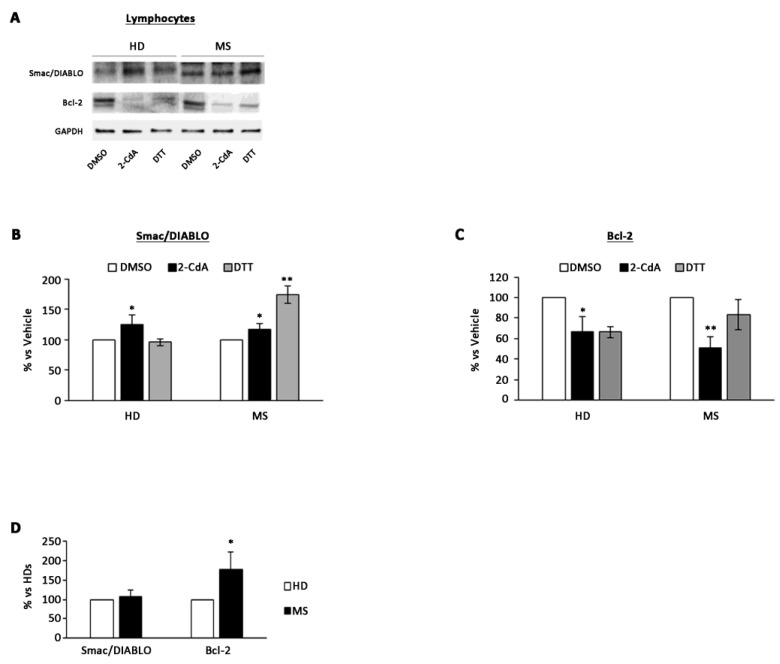
Effect of 2-CdA treatment on Smac/DIABLO and Bcl-2 protein expression in HDs and multiple sclerosis (MS) lymphocytes. (**A**) Representative Western blot of Smac/DIABLO and Bcl-2 levels performed on whole cell lysates. Bar graphs show quantification by densitometric analysis of Smac/DIABLO (**B**) and Bcl-2 (**C**) protein band normalized to GAPDH, used as loading control. Values (mean ± SEM of three independent experiments) are expressed as percentage of dimethyl sulfoxide (DMSO). (**D**) Bar graph showing densitometric analysis of Smac/DIABLO and Bcl-2 protein levels in healthy donors (HD) and multiple sclerosis (MS) lymphocytes not exposed to cladribine (2-CdA). Significance was calculated with Student’s t-test; * *p* < 0.05, ** *p* < 0.005 vs. DMSO (**B**,**C**) or vs. HDs (**C**). DTT, dithiothreitol.

There was a significant increase in pro-apoptotic condition in MS (20 ± 9%) and HD (25 ± 10%) lymphocytes exposed to 2-CdA (Figure 1A,B) compared with DMSO condition. Moreover, there was also a significant reduction of anti-apoptotic Bcl-2 protein level in both HD (34 ± 14%) and MS (50 ± 10%) lymphocytes exposed to 2-CdA (Figure 1A–C), and this reduction was more evident in MS lymphocytes (Figure 1C). Basal levels of anti-apoptotic protein Bcl-2 were significantly higher in MS lymphocytes compared with HDs (Figure 1D), whereas no differences were revealed for Smac/DIABLO protein. This evidence underlines the greater resistance of MS lymphocytes to apoptosis, which is subverted after exposure to 2-CdA (Figure 1C). Moreover, this is confirmed by pro-apoptotic protein level increase after 2-CdA exposure (Figure 1B).

**Figure 2:**
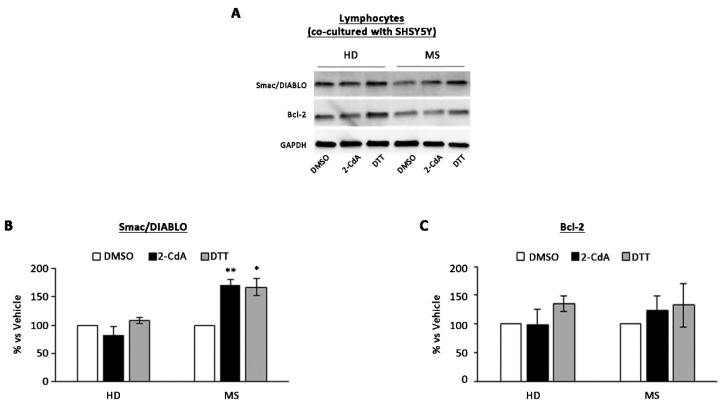
Effect of 2-CdA treatment on Smac/DIABLO and Bcl-2 protein expression in lymphocytes co-cultured with SH-SY5Y. (**A**) Representative Western blot of Smac/DIABLO and Bcl-2 levels performed on whole cell lysates. Bar graphs show quantification by densitometric analysis of Smac/DIABLO (**B**) and Bcl-2 (**C**) protein band normalized to GAPDH, used as loading control. Values (mean ± SEM of three independent experiments) are expressed as percentage of dimethyl sulfoxide (DMSO). Significance was calculated with Student’s t-test; * *p* < 0.05, ** *p* < 0.005 vs. DMSO. DTT, dithiothreitol.

The graph relative to densitometric analysis (Figure 2B,C), showed a significant increase (70 ± 20%) in pro-apoptotic condition in MS lymphocytes co-cultured with SH-SY5Y cells treated with 2-CdA (Figure 2A,B) compared with DMSO condition, whereas no significant reduction in anti-apoptotic Bcl-2 protein level was detected in both HD and MS co-cultured lymphocytes exposed to 2-CdA (Figure 2C).

**Figure 3:**
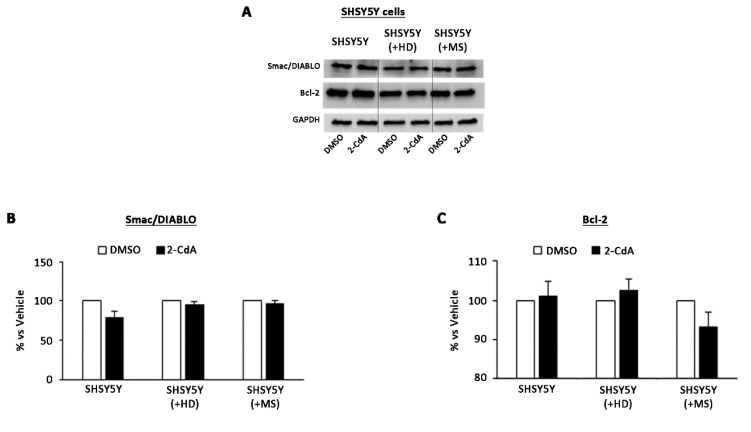
Effect of 2-CdA treatment on Smac/DIABLO and Bcl-2 protein expression in SH-SY5Y single-cultured and co-cultured with HD and MS lymphocytes. (**A**) Representative Western blot of Smac/DIABLO and Bcl-2 levels performed on whole cell lysates. Bar graphs show quantification by densitometric analysis of Smac/DIABLO (**B**) and Bcl-2 (**C**) protein band normalized to GAPDH, used as loading control. Values (mean ± SEM of three independent experiments) are expressed as percentage of dimethyl sulfoxide (DMSO). Significance was calculated with Student’s *t*-test vs. DMSO.

The most interesting aspect is that no differences were observed in terms of protein levels in SH-SY5Y cells exposed to 2-CdA in all the three conditions of culture: SH-SY5Y single-cultured and SH-SY5Y co-cultured with HD and MS lymphocytes (Figure 3).

These data confirm that 2-CdA could increase apoptotic condition in MS cells and does not affect neuronal cell apoptotic state because the apoptotic state induced by 2-CdA on lymphocytes does not occur in SH-SY5Y cells (Figure 3).

These results highlight the importance of 2-CdA as promising therapy able to increase the apoptotic condition in MS lymphocytes, preserving SH-SY5Y state.

### 3.2. Annexin V on SH-SY5Y Treated with 2-CdA

**Figure 4:**
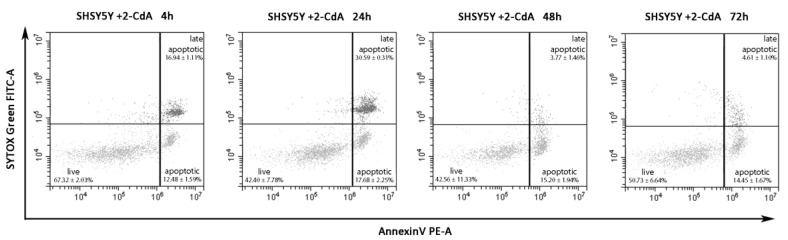
Representative dot plots of annexin V/SYTOX Green assay on SH-SY5Y cells exposed to 20 nM 2-CdA for 4, 24, 48, and 72 h. The population, gated on not treated events (control, not shown in dot plots) was divided into live events (**lower left**), apoptotic events (**lower right**), and late apoptotic events (**upper right**). The percentage of apoptotic events (**lower right**) was similar among the four time points analyzed: 12.48 ± 1.59% at 4 h; 17.68 ± 2.25% at 24 h; 15.20 ± 1.94% at 48 h; 14.45 ± 1.67% at 72 h (data are expressed in mean of percentage ± SD; *n* = 4).

The key effects of 2-CdA treatment are inhibition of cell proliferation and induction of apoptosis on lymphocytes. In this work, we wanted to evaluate a potential neuroprotective effect of 2-CdA. Flow cytometric experiments were conducted to evaluate the percentage of apoptotic cells exposed to 20 nM 2-CdA at different time points (Figure 4); not treated cells were used as negative controls, 20 nM DMSO was used as resuspending-drug medium control, and 2 mM DTT as positive control of cell death.

PS, which is normally restricted to the inner side of the bilayer in healthy cells, becomes externalized to the surface membrane of cells destined to die. This exposure allows the early recognition and phagocytosis of apoptotic cells, although PS externalization could be used as qualitative difference between the plasma membranes of apoptotic and viable cells. PS also exhibits procoagulant properties that are reversed by the binding of annexin V to PS [35]. The human vascular anticoagulant, annexin V, is a Ca^2+^-dependent phospholipid-binding protein that has a high affinity for PS. Annexin V labeled with a fluorophore can identify apoptotic cells by binding to PS exposed on the membrane surface [36]. Apoptotic cells become annexin V-positive after nuclear condensation has started, but before the cell has become permeable to nucleic acid binding dye (SYTOX Green).

**Figure 5:**
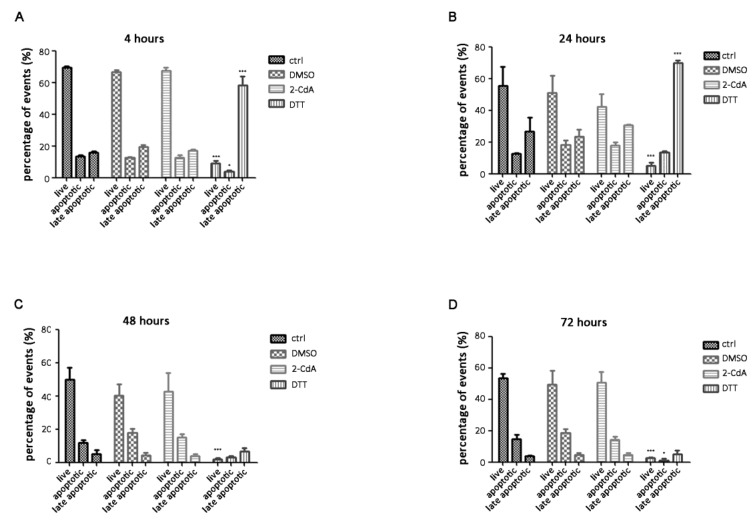
Histograms showing the percentage of live, apoptotic, and late apoptotic events at different timepoints. SH-SY5Y cells were analyzed by annexin V/SYTOX Green assay after exposure to 20 nM 2-CdA (2-CdA), dimethyl sulfoxide (DMSO), 20 mM dithiothreitol (DTT), and not treated cells (ctrl) for 4 (**A**), 24 (**B**), 48 (**C**), and 72 h (**D**). Data are expressed as mean ± SD and statistical analysis was computed using one-way ANOVA followed by the Newman–Keuls test; * *p* < 0.05, *** *p* < 0.001.

In these experiments, we wanted to focus on the percentage of apoptotic events revealed by flow cytometry on SH-SY5Y cells exposed to 2-CdA for 4, 24, 48, and 72 h, and data demonstrated that 2-CdA could not affect the apoptotic state of SH-SY5Y cells. In particular, the representative dot plots of SH-SY5Y cells exposed to 2-CdA in Figure 4 showed that the percentage of apoptotic events (lower right) was similar among the four time points analyzed: 12.48 ± 1.59% at 4 h; 17.68 ± 2.25% at 24 h; 15.20 ± 1.94% at 48 h; 14.45 ± 1.67% at 72 h; no statistical differences were detected between groups compared at different time points (Figure 5). These data demonstrate that neuron viability is not apparently impaired by 2-CdA in not only acute, but also protracted exposure of cells to 2-CdA (72 h).

Flow cytometric analysis showed that the percentage of apoptotic events was not statistically different among cells exposed to 2-CdA at different time points. The 2-CdA appeared to not affect the neuronal cell apoptotic state. Cells exposed to 2 mM DTT at 4 and 24 h were nearly all late apoptotic; at 48 and 72 h, the majority of cells were destroyed as debris. The significant differences were seen with 2 nM DTT treatment compared with the other corresponding groups of cells within the same time point (Figure 5).

Summing up, flow cytometric experiments on neuronal cells exposed to 2-CdA at different time points underline 2-CdA as a non-toxic “in vitro” therapy.

## 4. Discussion and Conclusions

Immunosuppressive drugs are of significant therapeutic importance in treating several autoimmune diseases. The 2-CdA is a deoxyadenosine analog prodrug that preferentially depletes lymphocytes, key cells underlying MS pathogenesis. It has been approved for the treatment of adults with highly active relapsing MS on the basis of data from pivotal clinical trials, including the phase 3 study CLARITY and its extension. Currently, no data on the effect on neurons are available.

In this in vitro study, we found that 2-CdA induced apoptosis in HDs’ and MS patients’ lymphocytes but not in SH-SY5Y neuronal cell line. At first, we showed increased apoptosis in MS lymphocytes during 2-CdA treatment compared with control cultures. Anti-apoptotic protein levels (Bcl-2) were reduced in HD lymphocytes under 2-CdA exposure, and this reduction was more evident in MS lymphocytes. The increase in apoptotic state (Smac/DIABLO levels) in MS lymphocytes exposed to 2-CdA compared with control cultures was also observed when they were cultured with a neuronal cell line. SH-SY5Y, alone or in co-cultures with lymphocytes, was not affected by drug exposure for apoptotic pattern, since pro-apoptotic protein Smac/DIABLO levels were not altered.

To further demonstrate that 2-CdA could not affect the apoptotic state of SH-SY5Y cells in particular, we conducted annexin V flow cytometry experiments on SH-SY5Y cells exposed to 2-CdA (Figure 4). Annexin V binding is one of the most commonly used assays to measure apoptosis [37]. When cells enter the apoptosis phase, PS, which is normally found on the inside of the cytoplasmic membrane, is found on the extracellular surface of the membrane, thus revealing annexin V-binding sites. The addition of dyes that are impermeant to live and apoptotic cells (such as SYTOX Green dye) allows subsequent stages of apoptosis and eventual cell death to be distinguished. Starting from this, we established that neuron viability seems to not be impaired by 2-CdA in not only acute, but also protracted exposure of cells to 2-CdA for 72 h (Figure 5).

We decided to choose SH-SY5Y cells, a human neuronal immortalized cell line, because it is widely used in cell culture models for providing insight into neuronal biology and has been largely used for in vitro studies to investigate neuronal toxicity, apoptotic mechanisms, and neuroimmune signaling between astrocytes and neurons [38] in many neurodegenerative diseases. A lot of Toll-like receptors (TLRs) such as TLR2, TLR3, TLR4, TLR7 and TLR8 are expressed in SH-SY5Y [39] similar to data obtained from in vivo studies or using primary neurons [40,41], demonstrating the same sensitivity to innate immunity and tissue injury and indicating that this cell line is appropriate for investigating innate immune functions. Moreover, it has been largely demonstrated that SH-SY5Y cells can be used to assess apoptosis activation. Many studies showed that dopamine induces apoptosis in SH-SY5Y cells primarily by activation of the p38 kinase and cytochrome c release, followed by caspase 9 and -3 activation [42]; Ouyang and colleagues demonstrated the critical role of the ASK1-p38/JNK signaling pathway in 6-OHDA (6-Hydroxydopamine)-induced apoptosis in SH-SY5Y cells [43]. It is well known that 6-OHDA is capable of reproducing the neuropathological and biochemical characteristics of Parkinson’s disease (PD) [44,45] and is commonly used to study the molecular signaling mechanisms involved in apoptotic cell death in SH-SY5Y cells [46]. Starting from these experiments, Ikeda et al. [47] have studied and demonstrated the mechanisms involved in the inhibition of apoptotic pathways on 6-OHDA-induced apoptosis in SH-SY5Y cells. Thus, on the basis of such evidence, this immortalized cell line proves useful into many contexts, as ours, in lymphocytes–neuronal interactions especially in searching a strategy for the treatment of neuronal disorder. Probably, the choice of this cellular line for apoptotic mechanisms studies under CdA stimulation could be a limit of our study since we should have used primary neuronal cells, such as human-induced pluripotent stem cells (iPSC), or astrocytes and/or oligodendrocytes, but the literature has encouraged us at first and, since we now use them in our laboratory for other studies, in the future we will want to explore soluble mediators released by neurons and peripheral mononuclear cells cocultures. In particular, we would focalize our attention on the production of pro-and anti-inflammatory cytokines or broadening our study to lymphocyte–glial cell interactions. In this context, some authors [33] investigated whether CdA was toxic to primary rat microglia, the resident macrophages in the brain, which are known to play a pivotal role during de- and regenerative processes in CNS diseases. Their studies suggested that CdA did not affect cell viability, and additionally, 2-CdA potentiated the gene expression of both anti- and pro-inflammatory molecules such as the anti-inflammatory TNF-R2. Therefore, in addition to its cytotoxicity on lymphocytes, these effects of CdA on microglia represent an additional possible mechanism that may exert the clinical effect. Due to its ability to pass the BBB, CdA may influence microglia even when there is a compartmentalization of the autoimmune reaction during the chronic progressive phase of MS [48].

In conclusion, data from the literature have already demonstrated the inhibitory effect of 2-CdA on the migratory capacity of immunocompetent cells across a BBB in vitro model [32], drawing attention to one of the key events in the immunopathogenesis of MS, in which both degenerative and immune processes are involved. Moreover, CdA effects on the microglia mentioned above might be due to some unknown mechanisms and represent slower effects of CdA in the CNS resident microglia as compared to peripheral-derived leukocytes. In this context, the importance of demonstrating the capacity of 2-CdA to modulate or prevent neuronal damage is of great importance now, supported by our data, focusing attention on the potential protective role of 2-CdA on the CNS where, during MS, inflammation seems to precede neurodegeneration. Our results, together with data from literature, suggest that 2-CdA could provide beneficial effects on resident CNS cells and significant effects in preventing damage to the BBB, contributing to its evaluation as a worthwhile and promising drug. Among the weaknesses of this paper, we acknowledge that we used a peripheral nervous system (PNS) cell line and that any conclusion about relevance to CNS disease needs to be further elucidated in other studies, although we are also conscious that PNS and CNS neurons may share apoptotic mechanisms, and the interaction between lymphocytes and neurons may be of relevance for other neurodegenerative diseases. Nevertheless, further in vivo studies, in a larger cohort of patients, need to confirm these findings.
